# Comparison of carbonic anhydrase-IX-targeted trifunctional radioligands between linear- and branched-chain arrangements

**DOI:** 10.3389/fnume.2025.1585027

**Published:** 2025-04-16

**Authors:** Kazuma Nakashima, Takayoshi Ichinose, Hiroyuki Watanabe, Masahiro Ono

**Affiliations:** Department of Patho-Functional Bioanalysis, Graduate School of Pharmaceutical Sciences, Kyoto University, Kyoto, Japan

**Keywords:** CA-IX, imidazothiadiazole sulfonamide, DOTA, albumin binder, linear-chain arrangement, branched-chain arrangement

## Abstract

**Background:**

Carbonic anhydrase-IX (CA-IX) is overexpressed in tumors due to hypoxic conditions and considered an attractive biomarker for tumor-targeting radioligands. The introduction of an albumin binder (ALB) to radioligands can delay their renal clearance, resulting in increased radioactivity delivered to tumors and decreased renal uptake of radioligands. In this study, we designed novel CA-IX-targeted trifunctional radioligands consisting of imidazothiadiazole sulfonamide (IS) as a CA-IX-targeted ligand, DOTA as a chelator with four free carboxylic groups, and lysine-conjugated 4-(*p*-iodophenyl)butyric acid (Lys-IPBA) as ALB, with IS-[^111^In]In-DOTADG-ALB in a linear-chain arrangement and [^111^In]In-DOTAGA-ALB-IS in a branched-chain arrangement. Fundamental properties of IS-[^111^In]In-DOTADG-ALB and [^111^In]In-DOTAGA-ALB-IS were evaluated by *in vitro* and *in vivo* assays.

**Methods:**

IS-DOTADG-ALB and DOTAGA-ALB-IS were synthesized and radiolabeled with [^111^In]InCl_3_. The stability of IS-[^111^In]In-DOTADG-ALB and [^111^In]In-DOTAGA-ALB-IS was evaluated by HPLC analysis after incubation in murine plasma. A cell saturation binding assay using CA-IX-positive HT-29 cells and albumin-binding assay were performed for IS-[^111^In]In-DOTADG-ALB and [^111^In]In-DOTAGA-ALB-IS to evaluate their capacity to bind CA-IX and albumin. Biodistribution assays of IS-[^111^In]In-DOTADG-ALB and [^111^In]In-DOTAGA-ALB-IS were performed using HT-29 tumor-bearing mice to evaluate their pharmacokinetics.

**Results:**

IS-[^111^In]In-DOTADG-ALB and [^111^In]In-DOTAGA-ALB-IS were successfully synthesized by ligand substitution reaction from their corresponding precursors. IS-[^111^In]In-DOTADG-ALB and [^111^In]In-DOTAGA-ALB-IS exhibited similar stabilities in murine plasma and affinities to CA-IX, although the affinities to albumin were higher for [^111^In]In-DOTAGA-ALB-IS compared with IS-[^111^In]In-DOTADG-ALB. In the biodistribution assays, [^111^In]In-DOTAGA-ALB-IS showed higher blood retention and tumor accumulation and lower renal uptake than IS-[^111^In]In-DOTADG-ALB, reflecting their albumin-binding affinities.

**Conclusion:**

These data suggest that the branched-chain arrangement of DOTAGA-ALB-IS may be useful for the design of CA-IX-targeted radioligands consisting of an IS ligand, DOTA, and Lys-IPBA.

## Introduction

Cancer is one of the most common diseases in humans, with an estimated 20 million new cases and 9.7 million cancer deaths worldwide in 2022 (1), accounting for a high proportion of deaths. Therefore, the development of techniques for cancer treatment has been strongly encouraged. Solid cancer cells rapidly proliferate prior to angiogenesis, forming hypoxic regions that are chronically deprived of oxygen supply from blood, a unique property not observed in normal tissues (2). Carbonic anhydrase-IX (CA-IX) is one of the most well-known biomarkers of cancer hypoxia (3–7). CA-IX catalyzes the reversible hydration of carbon dioxide to a bicarbonate anion and proton on membranes of tumor cells to regulate the intra- and extracellular pH for cell survival under hypoxic conditions (8). In the field of nuclear medicine, CA-IX-targeted radioligands have been reported based on low-molecular-weight inhibitors (9–12), peptides (13, 14), or monoclonal antibodies (15–18) for nuclear imaging and targeted radionuclide therapy. These ligands have expanded the utility of CA-IX-targeted radiotheranostics, which are used to tailor cancer therapy for individual patients.

However, most low-molecular-weight CA-IX-targeted radioligands are rapidly cleared from blood by glomerular filtration in the kidney, and show insufficient tumor retention or marked renal uptake of radioactivity (9–12), which may compromise tumor contrast in the process of diagnosis and increase the risk of nephrotoxicity in therapy. To overcome these challenges, the introduction of an albumin binder (ALB) to radioligands is considered a promising strategy (19). ALB is a low-molecular-weight molecule that interacts noncovalently with albumin in blood. Radioligands containing ALB can form high-molecular-weight complexes with albumin (∼66 kDa) in blood and show prolonged clearance from the kidney, resulting in increased tumor delivery and low renal uptake (20–24). In our previous study, a CA-IX-targeted ALB-containing trifunctional radioligand, IS-[^111^In]In-DO2A-ALB1, was developed based on our original CA-IX ligand, imidazothiadiazole sulfonamide (IS) (25). IS-[^111^In]In-DO2A-ALB1 was designed by combining an IS ligand, a macrocyclic chelator with two free carboxylic groups (DO2A), and lysine-conjugated 4-(*p*-iodophenyl)butyric acid (Lys-IPBA) as ALB in a linear-chain arrangement. IS-[^111^In]In-DO2A-ALB1 markedly increased tumor uptake and decreased renal uptake compared with a control radioligand without ALB, demonstrating the effectiveness of a strategy to introduce ALB into CA-IX-targeted radioligands.

**Figure 1:**
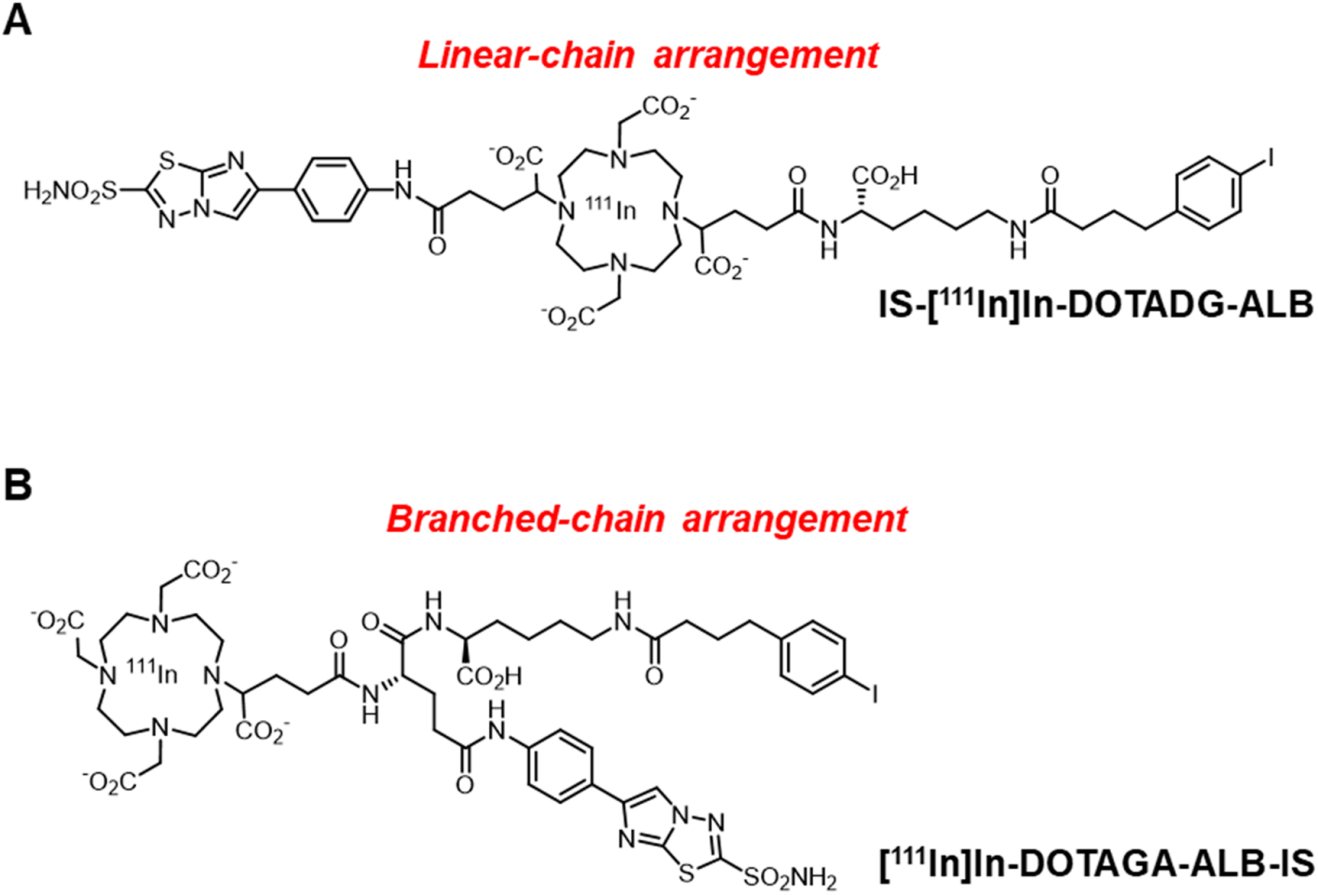
Chemical structures of IS-[^111^In]In-DOTADG-ALB in a linear-chain arrangement **(A)**, and [^111^In]In-DOTAGA-ALB-IS in a branched-chain arrangement **(B)**.

Trifunctional radioligands are generally designed with a linear- or branched-chain arrangement with a branched linker (26–29). However, there are no reports evaluating the effects of the molecular arrangement of CA-IX-targeted trifunctional radioligands on their properties. In this study, we aimed to compare the linear- or branched-chain arrangement to determine the most favorable design for CA-IX-targeted ALB-containing radioligands. DOTADG and DOTAGA, which are DOTA with four carboxylic groups, were selected because of their higher chelating properties than DO2A (30), and IS-DOTADG-ALB in a linear-chain arrangement (Figure 1A) and DOTAGA-ALB-IS in a branched-chain arrangement (Figure 1B) were newly designed as CA-IX-targeted trifunctional ligands. The properties of IS-[^111^In]In-DOTADG-ALB and [^111^In]In-DOTAGA-ALB-IS were evaluated and compared by *in vitro* and *in vivo* assays.

## Materials and methods

### General

All reagents were obtained commercially and used without further purification unless otherwise indicated. [^111^In]InCl_3_ was purchased from Nihon Medi-Physics (Tokyo, Japan). Low-resolution mass spectrometry (LRMS) was conducted with an LCMS-2020 system (Shimadzu, Kyoto, Japan), and high-resolution mass spectrometry (HRMS) was performed with a liquid chromatography/mass spectrometry ion trap time-of-flight (LCMS-IT-TOF) mass spectrometer (Shimadzu). Reversed-phase (RP)-HPLC was carried out using a Shimadzu system [LC-20AT or LC-20AD pump with an SPD-20A ultraviolet (UV) detector, *λ* = 220 or 254 nm] with a Cosmosil C_18_ column (5C_18_-AR-II, 4.6 × 150 or 10 × 250 mm; Nacalai Tesque, Kyoto, Japan).

### Chemistry

4-(4,10-Bis[2-(*tert*-butoxy)-2-oxoethyl]-7-(1-(*tert*-butoxy)-5-(((S)-1-(*tert*-butoxy)-6-[4-(4-iodophenyl)butanamido]-1-oxohexan-2-yl)amino)-1,5-dioxopentan-2-yl)-1,4,7,10-tetraazacyclododecan-1-yl)-5-(*tert*-butoxy)-5-oxopentanoic Acid (**1**).

Compound **1** was synthesized according to our previous report (31).

6-(4-Aminophenyl)imidazo[2,1-*b*][1,3,4]thiadiazole-2-sulfonamide (**2**).

Compound **2** was synthesized according to our previous report (32).

Di-*tert*-butyl-2,2'-(4-(1-(*tert*-butoxy)-1,5-dioxo-5-((4-[2-sulfamoylimidazo[2,1-*b*][1,3,4]thiadiazol-6-yl]phenyl)amino)pentan-2-yl)-10-(1-(*tert*-butoxy)-5-(((*S*)-1-(*tert*-butoxy)-6-[4-(4-iodophenyl)butanamido]-1-oxohexan-2-yl)amino)-1,5-dioxopentan-2-yl)-1,4,7,10-tetraazacyclododecane-1,7-diyl)diacetate (**3**).

To a solution of **1** (130 mg, 0.11 mmol) in anhydrous *N*,*N*-dimethylformamide (DMF) (400 µl) were added 1-[1-(cyano-2-ethoxy-2-oxo-ethylideneaminooxy)-dimethylaminomorpholino]uronium hexafluorophosphate (COMU) (181 mg, 0.42 mmol) and *N*,*N*-diisopropylethylamine (DIPEA) (60 µl, 0.42 mmol) at 0°C. The mixture was stirred at 0°C for 10 min, and **2** (125 mg, 0.42 mmol) was added to it at 0°C. After being stirred at room temperature for 20 h, the mixture was purified by RP-HPLC on a Cosmosil C_18_ column (5C_18_-AR-II, 10 × 250 mm) using a mobile phase [H_2_O with 0.1% trifluoroacetic acid (TFA)/MeCN with 0.1% TFA = 70/30 (0 min) to 30/70 (40 min)], which was delivered at a flow rate of 4.0 ml/min, to give 76 mg of **3** (48% yield). LRMS [electrospray ionization (ESI)] *m/z* calcd for C_68_H_105_IN_11_O_15_S_2_^+^, 1,506.6 [M + H]^+^; found, 1,506.4.

2,2'-(4-(1-Carboxy-4-(((*S*)-1-carboxy-5-[4-(4-iodophenyl)butanamido]pentyl)amino)-4-oxobutyl)-10-(1-carboxy-4-oxo-4-((4-[2-sulfamoylimidazo[2,1-*b*][1,3,4]thiadiazol-6-yl]phenyl)amino)butyl)-1,4,7,10-tetraazacyclododecane-1,7-diyl)diacetic Acid (IS-DOTADG-ALB) (**4**).

Compound **3** (76 mg, 51 µmol) was dissolved in a cocktail of TFA (1.88 ml), H_2_O (50 µl), 1,2-ethanedithiol (50 µl), and triisopropylsilane (TIPS) (20 µl). After being stirred at room temperature for 7 h, the solution was evaporated, and the residue was purified by RP-HPLC performed with a Cosmosil C_18_ column (5C_18_-AR-II, 10 × 250 mm) using a mobile phase [H_2_O with 0.1% TFA/MeCN with 0.1% TFA = 90/10 (0 min) to 10/90 (40 min)], which was delivered at a flow rate of 4.0 ml/min, to give 12 mg of **4** (19% yield). HRMS (ESI) *m/z* calcd for C_48_H_65_IN_11_O_15_S_2_^+^, 1,226.3143 [M + H]^+^; found, 1,226.3152.

Indium (III) 2,2'-(4-(1-Carboxy-4-(((*S*)-1-carboxy-5-[4-(4-iodophenyl)butanamido]pentyl)amino)-4-oxobutyl)-10-(1-carboxy-4-oxo-4-((4-[2-sulfamoylimidazo[2,1-*b*][1,3,4]thiadiazol-6-yl]phenyl)amino)butyl)-1,4,7,10-tetraazacyclododecane-1,7-diyl)diacetic Acid (IS-[^nat^In]In-DOTADG-ALB) (**5**).

To a solution of **4** (1.0 mg, 0.82 µmol) in a cocktail of MeCN (330 µl) and acetate buffer (0.1 M, pH 6.0, 330 µl) was added anhydrous indium (III) chloride (1.8 mg, 15.7 µmol). The mixture was incubated at 90°C for 20 min, centrifuged, and the supernatant was purified by RP-HPLC performed with a Cosmosil C_18_ column (5C_18_-AR-II, 4.6 × 150 mm) using a mobile phase [H_2_O with 0.1% TFA/MeCN with 0.1% TFA = 70/30 (0 min) to 30/70 (40 min)], which was delivered at a flow rate of 1 ml/min, to give **5**. The obtained amount of **5** was too small to calculate the yield accurately. LRMS (ESI) *m/z* calcd for C_48_H_62_IInN_11_O_15_S_2_^+^, 1,338.2 [M + H]^+^; found, 1,338.0.

*tert*-Butyl-*N*^6^-[4-(4-iodophenyl)butanoyl]-_L_-lysinate (**6**).

Compound **6** was synthesized according to our previous report (31).

*tert*-Butyl-*N*^2^-((*S*)-2-amino-5-oxo-5-[(2-phenylpropan-2-yl)oxy]pentanoyl)-*N*^6^-[4-(4-iodophenyl)butanoyl]-_L_-lysinate (**7**).

To a solution of 2-({[(9*H*-fluoren-9-yl)methoxy]carbonyl}amino)-5-oxo-5-[(2-phenylpropan-2-yl)oxy]pentanoic acid (257 mg, 0.53 mmol) in anhydrous DMF (3.0 ml) were added 1-ethyl-3-(dimethylaminopropyl)carbodiimide hydrochloride (EDC·HCl) (101 mg, 0.53 mmol), 1-hydroxy-7-azabenzotriazole (HOAt) (72 mg, 0.53 mmol), and triethylamine (73 µl, 0.53 mmol) at 0°C. The mixture was stirred at 0°C for 10 min, and **6** (250 mg, 0.53 mmol) was added to it at 0°C. The mixture was stirred at room temperature for 24 h, and piperidine (750 µl) was added to it. After being stirred at room temperature for 22 h, the mixture was mixed with H_2_O and extracted with ethyl acetate. The organic layer was washed with brine and dried over sodium sulfate, the mixture was filtrated, and the filtrate was evaporated. The residue was purified by silica gel chromatography (chloroform/methanol = 91/9) to give 353 mg of **7** (93% yield). LRMS (ESI) *m/z* calcd for C_34_H_49_IN_3_O_6_^+^, 722.3 [M + H]^+^; found, 722.2.

5-(*tert*-Butoxy)-5-oxo-4-{4,7,10-tris[2-(*tert*-butoxy)-2-oxoethyl]-1,4,7,10-tetraazacyclododecan-1-yl}pentanoic Acid (**8**).

Compound **8** was synthesized according to our previous report (33).

Tri-*tert*-butyl-2,2’,2''-(10-((10*S*,13*S*)-13-(*tert*-butoxycarbonyl)-22-(4-iodophenyl)-2,2-dimethyl-4,8,11,19-tetraoxo-10-{3-oxo-3-[(2-phenylpropan-2-yl)oxy]propyl}-3-oxa-9,12,18-triazadocosan-5-yl)-1,4,7,10-tetraazacyclododecane-1,4,7-triyl)triacetate (**9**).

To a solution of **8** (790 mg, 1.13 mmol) in anhydrous DMF (700 µl) were added EDC·HCl (108 mg, 0.56 mmol), HOAt (77 mg, 0.56 mmol), and triethylamine (78 µl, 0.56 mmol) at 0°C. The mixture was stirred at 0°C for 10 min, and **7** (203 mg, 0.28 mmol) was added to it at 0°C. After being stirred at room temperature for 48 h, the mixture was mixed with H_2_O and extracted with chloroform. The organic layer was washed with brine and dried over sodium sulfate, the mixture was filtrated, and the filtrate was evaporated. The residue was purified by silica gel chromatography (chloroform/methanol = 82/18) to give 350 mg of **9** (88% yield). LRMS (ESI) *m/z* calcd for C_69_H_112_IN_7_O_15_^2+^, 702.9 [M + 2H]^2+^; found, 702.9.

Tri-*tert*-butyl-2,2’,2''-(10-((10*S*,13*S*)-13-(*tert*-butoxycarbonyl)-22-(4-iodophenyl)-2,2-dimethyl-4,8,11,19-tetraoxo-10-(3-oxo-3-((4-[2-sulfamoylimidazo[2,1-*b*][1,3,4]thiadiazol-6-yl]phenyl)amino)propyl)-3-oxa-9,12,18-triazadocosan-5-yl)-1,4,7,10-tetraazacyclododecane-1,4,7-triyl)triacetate (**10**).

Compound **9** (350 mg, 0.25 mmol) was dissolved in a cocktail of TFA (140 µl), dichloromethane (DCM) (1.72 ml), and TIPS (140 µl). After being stirred at room temperature for 3 h, the solution was evaporated. The residue was dissolved in anhydrous DMF (700 µl), and EDC·HCl (74 mg, 0.54 mmol), HOAt (104 mg, 0.54 mmol), and triethylamine (55 mg, 0.54 mmol) were added to the mixture at 0°C. The mixture was stirred at 0°C for 10 min, and **2** (161 mg, 0.54 mmol) was added to it at 0°C. After being stirred at room temperature for 12 h, the mixture was evaporated, and the residue was purified by RP-HPLC performed with a Cosmosil C_18_ column (5C_18_-AR-II, 10 × 250 mm) using a mobile phase [H_2_O with 0.1% TFA/MeCN with 0.1% TFA = 70/30 (0 min) to 30/70 (40 min)], which was delivered at a flow rate of 4 ml/min, to give 107 mg of **10** (27% yield). HRMS (ESI) *m/z* calcd for C_70_H_109_IN_12_O_16_S_2_^2+^, 782.3280 [M + 2H]^2+^; found, 782.3263.

2,2’,2''-(10-(1-Carboxy-4-(((*S*)-1-(((*S*)-1-carboxy-5-[4-(4-iodophenyl)butanamido]pentyl)amino)-1,5-dioxo-5-((4-[2-sulfamoylimidazo[2,1-*b*][1,3,4]thiadiazol-6-yl]phenyl)amino)pentan-2-yl)amino)-4-oxobutyl)-1,4,7,10-tetraazacyclododecane-1,4,7-triyl)triacetic Acid (DOTAGA-ALB-IS) (**11**).

Compound **10** (100 mg, 64 µmol) was dissolved in a cocktail of TFA (1.88 ml), H_2_O (50 µl), 1,2-ethanedithiol (50 µl), and TIPS (20 µl). After being stirred at room temperature for 9 h, the solution was evaporated, and the residue was purified by RP-HPLC performed with a Cosmosil C_18_ column (5C_18_-AR-II, 10 × 250 mm) using a mobile phase [H_2_O with 0.1% TFA/MeCN with 0.1% TFA = 70/30 (0 min) to 30/70 (40 min)], which was delivered at a flow rate of 4 ml/min, to give **11**. HRMS (ESI) *m/z* calcd for C_50_H_69_IN_12_O_16_S_2_^2+^, 642.1715 [M + 2H]^2+^; found, 642.1739.

Indium (III) 2,2’,2''-(10-(1-Carboxy-4-(((*S*)-1-(((*S*)-1-carboxy-5-[4-(4-iodophenyl)butanamido]pentyl)amino)-1,5-dioxo-5-((4-[2-sulfamoylimidazo[2,1-*b*][1,3,4]thiadiazol-6-yl]phenyl)amino)pentan-2-yl)amino)-4-oxobutyl)-1,4,7,10-tetraazacyclododecane-1,4,7-triyl)triacetic Acid ([^nat^In]In-DOTAGA-ALB-IS) (**12**).

To a solution of **11** (2.8 mg, 2.18 µmol) in a cocktail of MeCN (150 µl) and H_2_O (150 µl) was added anhydrous indium (III) chloride (7.5 mg, 65.5 µmol). The mixture was incubated at 90°C for 30 min, centrifuged, and the supernatant was purified by RP-HPLC performed with a Cosmosil C_18_ column (5C_18_-AR-II, 4.6 × 150 mm) using a mobile phase [H_2_O with 0.1% TFA/MeCN with 0.1% TFA = 70/30 (0 min) to 30/70 (40 min)], which was delivered at a flow rate of 1 ml/min. LRMS (ESI) *m/z* calcd for C_50_H_66_IInN_12_O_14_S_2_^2+^, 698.1 [M + 2H]^2+^; found, 698.2.

### Radiolabeling

A sodium acetate buffer (0.1 M, pH 5.9, 100–480 μl) was mixed with the same volume of [^111^In]InCl_3_ solution (2.4–22 MBq), and then IS-DOTADG-ALB or DOTAGA-ALB-IS (1 µg/µl in DMSO, 1.0–2.4 μl) was added to the mixture. The mixture was incubated at 90°C for 20 min and purified by RP-HPLC with a Cosmosil C_18_ column (5C_18_-AR-II, 4.6 × 150 mm) using a mobile phase [H_2_O with 0.1% TFA/MeCN with 0.1% TFA = 70/30 (0 min) to 30/70 (40 min)], which was delivered at a flow rate of 1 ml/min. The HPLC solvent was removed using N_2_ or Ar gas flow, and obtained IS-[^111^In]In-DOTADG-ALB (4.7–20.5 GBq/μmol) and [^111^In]In-DOTAGA-ALB-IS (3.8–7.1 GBq/μmol) were used for *in vitro* and *in vivo* assays.

### Animals

All animal experiments were performed in accordance with our institutional guidelines and approved by the Kyoto University Animal Care Committee. ddY and BALB/c *nu*/*nu* mice (5 weeks old) were purchased from Japan SLC (Shizuoka, Japan). The animals were housed in a sterile environment under a 12-h light−dark cycle, fed standard chow, and had free access to water.

### Cell culture

HT-29 and MDA-MB-231, which are human colorectal cancer and human breast cancer cell lines, respectively, were purchased from Sumitomo Dainippon Pharma (Osaka, Japan). The cells were maintained in Dulbecco's modified Eagle's medium (DMEM; Nacalai Tesque) supplemented with 10% heat-inactivated fetal bovine serum (Thermo Fisher Scientific, Massachusetts, USA) and 100 U/ml of penicillin and streptomycin at 37°C in an atmosphere containing 5% CO_2_.

### *In vitro* stability assay in murine plasma

Fresh ddY mouse blood was collected in venous blood collection tubes (Becton, Dickinson and Company, New Jersey, U.S.A.). The blood was centrifuged at 3,000 *g* for 10 min to obtain murine plasma. To the fresh plasma (200 μl) was added IS-[^111^In]In-DOTADG-ALB or [^111^In]In-DOTAGA-ALB-IS (436–544 kBq) in aqueous solution (15 μl). The mixture was incubated at 37°C for 24 h (*n* = 3), and MeCN (250 μl) was added to it. The mixture was then centrifuged at 10,000 *g* for 5 min, and the supernatant was analyzed by RP-HPLC under the same conditions as in radiolabeling.

### *In vitro* cell-binding assay

HT-29 and MDA-MB-231 cells were incubated in 12-well plates (2 × 10^5^ cells/well) at 37°C in an atmosphere containing 5% CO_2_ for 48 h. After removing the medium, IS-[^111^In]In-DOTADG-ALB (37 kBq, 1.8–3.2 pmol, 11.6–20.5 GBq/μmol) or [^111^In]In-DOTAGA-ALB-IS (37 kBq, 5.8–9.7 pmol, 3.8–6.3 GBq/μmol) in DMEM (1 ml) with or without acetazolamide (50 μM) was added to each well, and the plates were incubated at 37°C in an atmosphere containing 5% CO_2_ for 2 h. After incubation, the wells were rinsed with PBS (pH 7.4, 1 ml), and the cells were lysed with 1 N NaOH aqueous solution (200 μl × 2). The radioactivity of the cell solution was measured with a γ-counter (2470 WIZARD^2^; PerkinElmer, Massachusetts, U.S.A.). The protein concentration of the cell solution was determined using a bicinchoninic acid protein assay kit (Thermo Fisher Scientific).

### Cell saturation binding assay

A cell saturation binding assay was performed according to a previous report (34). HT-29 cells were incubated in 12-well plates (2 × 10^5^ cells/well) at 37°C in an atmosphere containing 5% CO_2_ for 48 h. After removal of the medium, the cells were washed with fresh DMEM (1 ml). Subsequently, they were incubated with increasing concentrations of IS-[^111^In]In-DOTADG-ALB or [^111^In]In-DOTAGA-ALB-IS (0.39–200 nM, 3.91–4.69 MBq/nmol) in DMEM with or without acetazolamide (100 μM), respectively, at 4°C for 1 h. After removal of the medium, the cells were washed with DMEM (1 ml × 2) and lysed with 1 N NaOH aqueous solution (200 μl × 2). The radioactivity of the cell solution was measured with a *γ*-counter (2470 WIZARD^2^). Total protein was determined using the bicinchoninic acid protein assay kit.

### *In vitro* albumin-binding assay

An albumin-binding assay was performed according to previous reports (35, 36). IS-[^111^In]In-DOTADG-ALB or [^111^In]In-DOTAGA-ALB-IS (2.23–4.66 MBq/nmol, 50 kBq) was incubated with HSA solutions in PBS (0.3 μM–3 mM, 150 μl) at 37°C for 30 min. The mixture was centrifuged at 14,000 g for 30 min on Amicon Ultra 0.5 ml (10 kDa, Merck Millipore, Massachusetts, U.S.A.). The inserts of filter devices were inverted and centrifuged at 200 *g* for 3 min. The radioactivity of the protein fraction (A_protein_), filtrate (A_filtrate_), and filter unit (A_filter_) was measured using a *γ*-counter (2470 WIZARD^2^). The binding ratio to HSA (%) was calculated as [A_protein_/(A_protein_ + A_filtrate_+A_filter_) × 100]. The binding ratio was plotted against HSA-to-ligand concentration ratios and fitted with a nonlinear regression curve using GraphPad Prism software (version 6.0) to obtain the inverse half-maximum binding (B_50_) values. Relative binding affinities were evaluated with the inverse ratio of the B_50_ value of each compound, and the affinity of IS-[^111^In]In-DOTADG-ALB was set to 1.00 as a reference.

### Tumor model

Under anesthesia (induced with 2% isoflurane), BALB/c *nu*/*nu* mice (female, 5 weeks old) were subcutaneously inoculated with HT-29 cells (5 × 10^6^ cells/mouse) in 150 μl of a mixture of DMEM and Matrigel (Corning, Arizona, U.S.A.) at a ratio of 1:1, in the right flank. HT-29 tumors were grown for 2 weeks to enable them to reach 0.8 cm in diameter.

### Biodistribution assay using model mice

A saline solution (100 μl) of IS-[^111^In]In-DOTADG-ALB (259 kBq, 55 pmol, 4.7 GBq/μmol) or [^111^In]In-DOTAGA-ALB-IS (259 kBq, 37–48 pmol, 5.4–7.0 GBq/μmol) was injected into the tail vein of HT-29 tumor-bearing mice. At 1, 4, 24, 48, 96, and 192 h postinjection (p.i.), the mice (*n* = 4) were euthanized. Blood, organs, and tissues of interest were collected and weighed, and the radioactivity of the collected samples was measured with a γ-counter (2480 WIZARD^2^; PerkinElmer).

### Statistical analysis

All data were analyzed with GraphPad Prism software (version 6.0) or Microsoft Excel.

## Results and discussion

### Synthesis

**Scheme 1:**
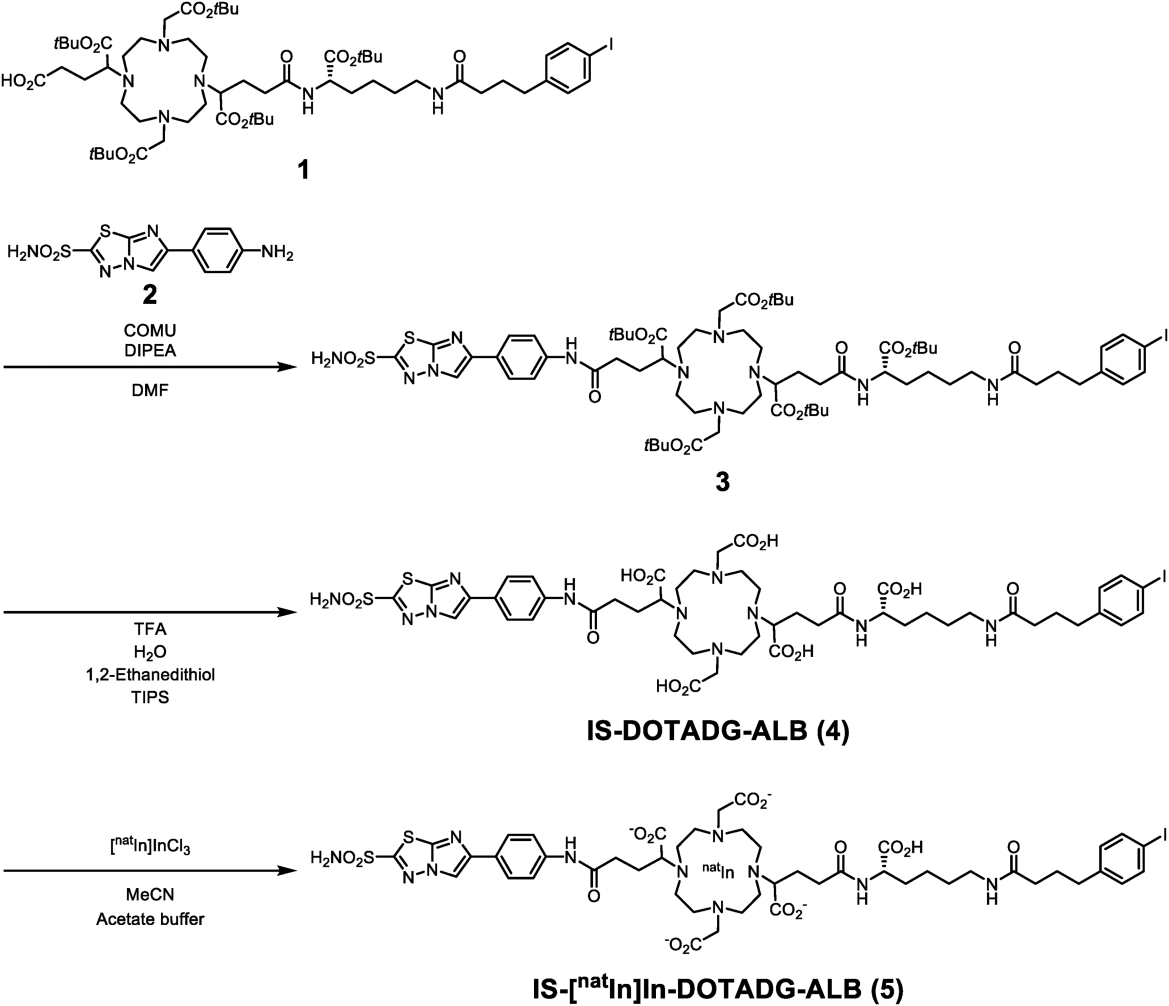
Synthetic routes for IS-[^nat^In]In-DOTADG-ALB.

**Scheme 2:**
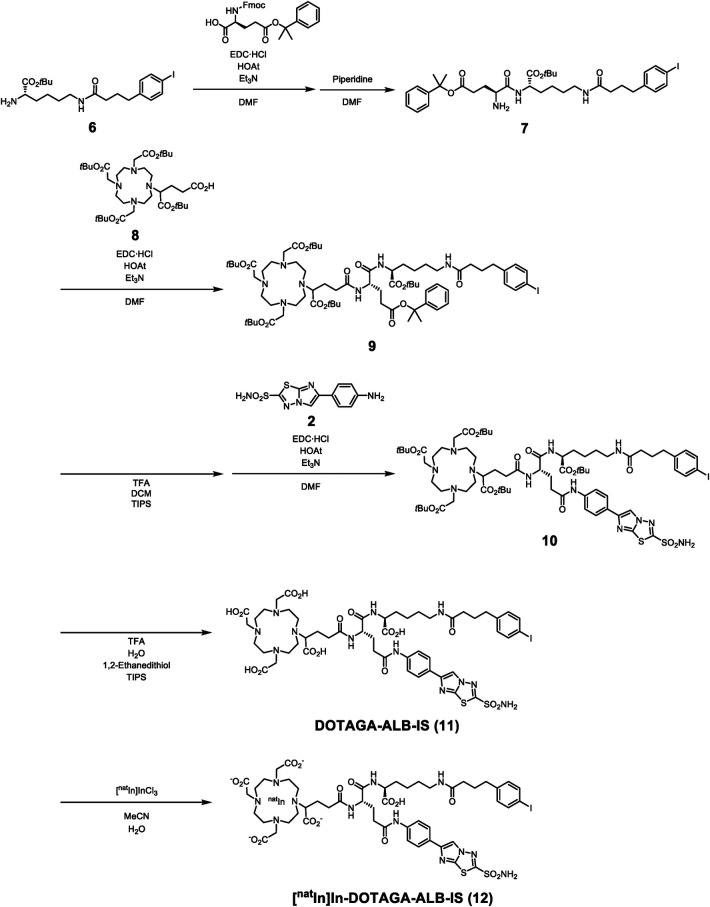
Synthetic routes for [^nat^In]In-DOTAGA-ALB-IS.

Synthetic routes for IS-DOTADG-ALB and DOTAGA-ALB-IS are described in Schemes 1, 2. IS-DOTADG-ALB (**4**) was synthesized from a starting material containing both DOTADG and Lys-IPBA (**1**) by condensation reaction with an IS ligand (**2**) in a linear-chain arrangement and subsequent deprotection reaction for *t*Bu-protected carboxylic groups. DOTAGA-ALB-IS (**11**) was synthesized from a branched linker containing Lys-IPBA (**7**) by consecutive condensation reactions with DOTAGA (**8**) and an IS ligand (**2**) followed by *t*Bu-deprotection. IS-[^nat^In]In-DOTADG-ALB (**5**) and [^nat^In]In-DOTAGA-ALB-IS (**12**) were synthesized from corresponding precursors by a ligand substitution reaction under acidic conditions.

### Radiolabeling

^111^In-labeling of IS-DOTADG-ALB and DOTAGA-ALB-IS was performed by heating at 90°C in acetate buffer solution. The ^111^In-chelation reaction proceeded with favorable radiochemical yields of 74.3% for IS-[^111^In]In-DOTADG-ALB (Figure S1) and 72.8% for [^111^In]In-DOTAGA-ALB-IS (Figure S2). The obtained IS-[^111^In]In-DOTADG-ALB and [^111^In]In-DOTAGA-ALB-IS showed high radiochemical purities (RCPs) of >95% by RP-HPLC analysis and their retention times matched those of nonradioactive IS-[^nat^In]In-DOTADG-ALB (Figure S3) and [^nat^In]In-DOTAGA-ALB-IS (Figure S4). These data indicate that the desired radioligands, IS-[^111^In]In-DOTADG-ALB and [^111^In]In-DOTAGA-ALB-IS, were obtained with high purities. Moreover, the ^111^In-labeling of IS-DO2A-ALB was performed at a concentration of approximately 32 μM and the radiochemical yield was 31–44% (25), although IS-[^111^In]In-DOTADG-ALB and [^111^In]In-DOTAGA-ALB-IS were synthesized at a concentration of approximately 3.2 μM and the radiochemical yields were 73–74%. These data suggested that the replacement of DO2A with DOTA improved the radiolabeling efficacy and radiochemical yield of IS-based ^111^In-labeled ligands.

### *In vitro* stability assay

**Figure 2:**
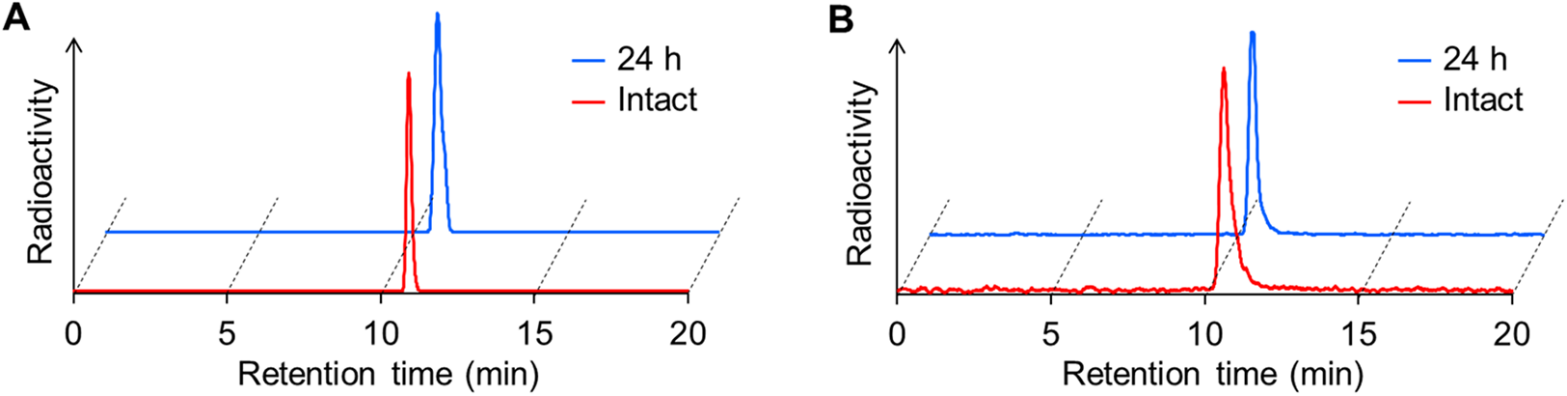
*In vitro* stability of IS-[^111^In]In-DOTADG-ALB **(A)** and [^111^In]In-DOTAGA-ALB-IS **(B)** after incubation in murine plasma for 24 h. RCPs of radioligands were determined by RP-HPLC with a Cosmosil C_18_ column (5C_18_-AR-II, 4.6 × 150 mm) using a mobile phase [H_2_O with 0.1% TFA/MeCN with 0.1% TFA = 70/30 (0 min) to 30/70 (40 min)], which was delivered at a flow rate of 1 ml/min.

The stability of IS-[^111^In]In-DOTADG-ALB and [^111^In]In-DOTAGA-ALB-IS was evaluated using murine plasma. After the radioligands were incubated for 24 h in murine plasma at 37°C, their RCPs were determined to be over 95% by RP-HPLC analysis (Figure 2). These results indicate that the difference in arrangement of linear and branched chains has little effect on the stabilities of CA-IX-targeted radioligands.

### *In vitro* assays using tumor cells

**Figure 3:**
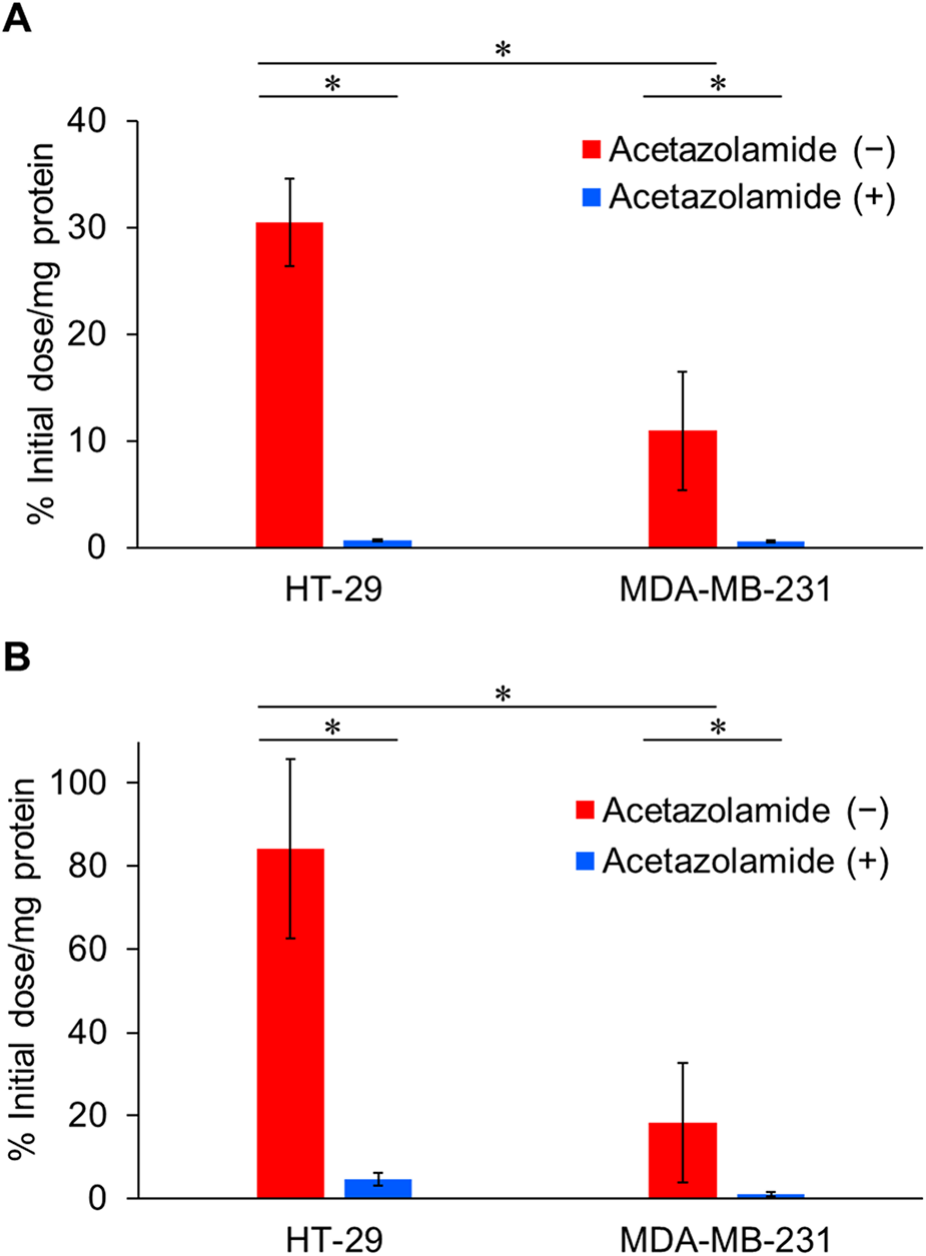
Binding of IS-[^111^In]In-DOTADG-ALB **(A)** and [^111^In]In-DOTAGA-ALB-IS **(B)** to HT-29 (CA-IX high expression) and MDA-MB-231 (CA-IX low expression) cells. Values are expressed as the mean ± standard deviation of nine independent experiments. *P < 0.05 [one-way analysis of variance (ANOVA) with *post-hoc* Tukey's test].

The CA-IX specificities of IS-[^111^In]In-DOTADG-ALB and [^111^In]In-DOTAGA-ALB-IS were assessed by a cell binding assay using HT-29 and MDA-MB-231 cells with high and low CA-IX-expression levels, respectively (10). IS-[^111^In]In-DOTADG-ALB and [^111^In]In-DOTAGA-ALB-IS showed marked binding to HT-29 cells (30.5 ± 4.1 and 84.1 ± 21.6% initial dose/mg protein, respectively) (Figure 3). The binding of IS-[^111^In]In-DOTADG-ALB and [^111^In]In-DOTAGA-ALB-IS to MDA-MB-231 cells was significantly lower (10.9 ± 5.6 and 18.2 ± 14.5% initial dose/mg protein, respectively) than that to HT-29 cells. Moreover, the binding to HT-29 and MDA-MB-231 cells was significantly decreased by the addition of acetazolamide, a classical CA inhibitor, for both IS-[^111^In]In-DOTADG-ALB (0.65 ± 0.12 and 0.56 ± 0.10% initial dose/mg protein, respectively) and [^111^In]In-DOTAGA-ALB-IS (4.61 ± 1.46 and 1.01 ± 0.54% initial dose/mg protein, respectively). These results of the cell binding assay indicate CA-IX specificities of IS-[^111^In]In-DOTADG-ALB and [^111^In]In-DOTAGA-ALB-IS.

**Figure 4:**
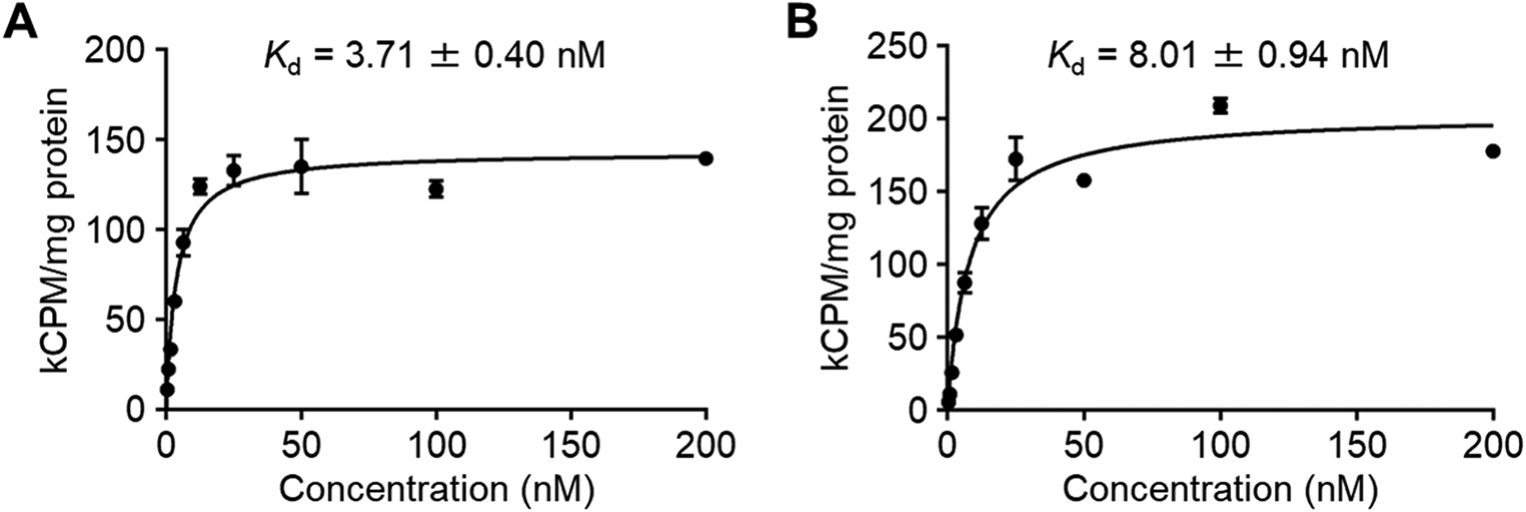
The specific binding of IS-[^111^In]In-DOTADG-ALB **(A)** and [^111^In]In-DOTAGA-ALB-IS **(B)** to HT-29 cells in the cell saturation binding assay.

The affinity of the radioligands to CA-IX was evaluated by a cell saturation binding assay using HT-29 cells. The *K*_d_ values of IS-[^111^In]In-DOTADG-ALB and [^111^In]In-DOTAGA-ALB-IS to HT-29 cells were determined as 3.71 ± 0.40 and 8.01 ± 0.94 nM, respectively (Figure 4). These *K*_d_ values were low and no marked differences were observed between them, suggesting that the CA-IX-binding properties of IS-[^111^In]In-DOTADG-ALB and [^111^In]In-DOTAGA-ALB-IS were little affected by arrangements of the IS ligand, DOTA, and Lys-IPBA.

### *In vitro* albumin-binding assay

**Figure 5:**
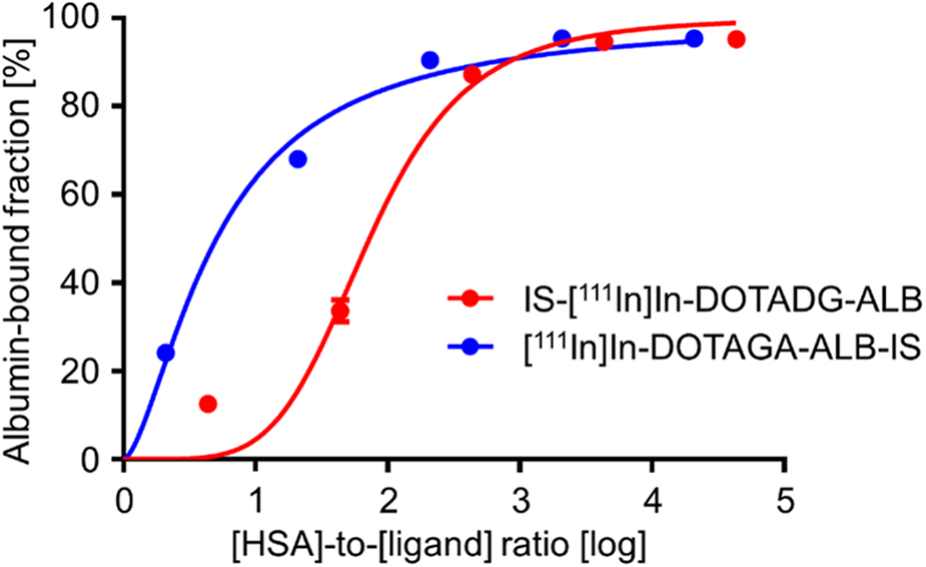
Albumin-binding curves of IS-[^111^In]In-DOTADG-ALB and [^111^In]In-DOTAGA-ALB-IS using HSA. Data are expressed as the mean ± standard deviation of three independent experiments.

The albumin-binding properties of the radioligands were assessed using serum albumin. The albumin-bound fraction of IS-[^111^In]In-DOTADG-ALB and [^111^In]In-DOTAGA-ALB-IS was increased with an increasing ratio of HSA to the ligand concentration (Figure 5), suggesting the albumin-binding properties of IS-[^111^In]In-DOTADG-ALB and [^111^In]In-DOTAGA-ALB-IS. Moreover, we calculated the relative binding affinity of the radioligands to albumin with the inverse ratio of the half-maximum binding (B_50_) value. When the relative binding affinity of IS-[^111^In]In-DOTADG-ALB was set as 1.00 as a reference, that of [^111^In]In-DOTAGA-ALB-IS was calculated as 14.4, indicating the higher albumin-binding affinity of [^111^In]In-DOTAGA-ALB-IS than that of IS-[^111^In]In-DOTADG-ALB. The arrangement of DOTA in the radioligands is unlikely to affect their albumin-binding properties because the distance between DOTA and Lys-IPBA is mostly the same between IS-[^111^In]In-DOTADG-ALB and [^111^In]In-DOTAGA-ALB-IS; therefore, the position of the IS ligand in radioligands is considered to affect their albumin-binding properties. These findings suggest that the arrangement of the IS ligand, DOTA, and Lys-IPBA may be important for IS-based radioligands in terms of altering albumin-binding properties.

### Biodistribution assay

**Figure 6:**
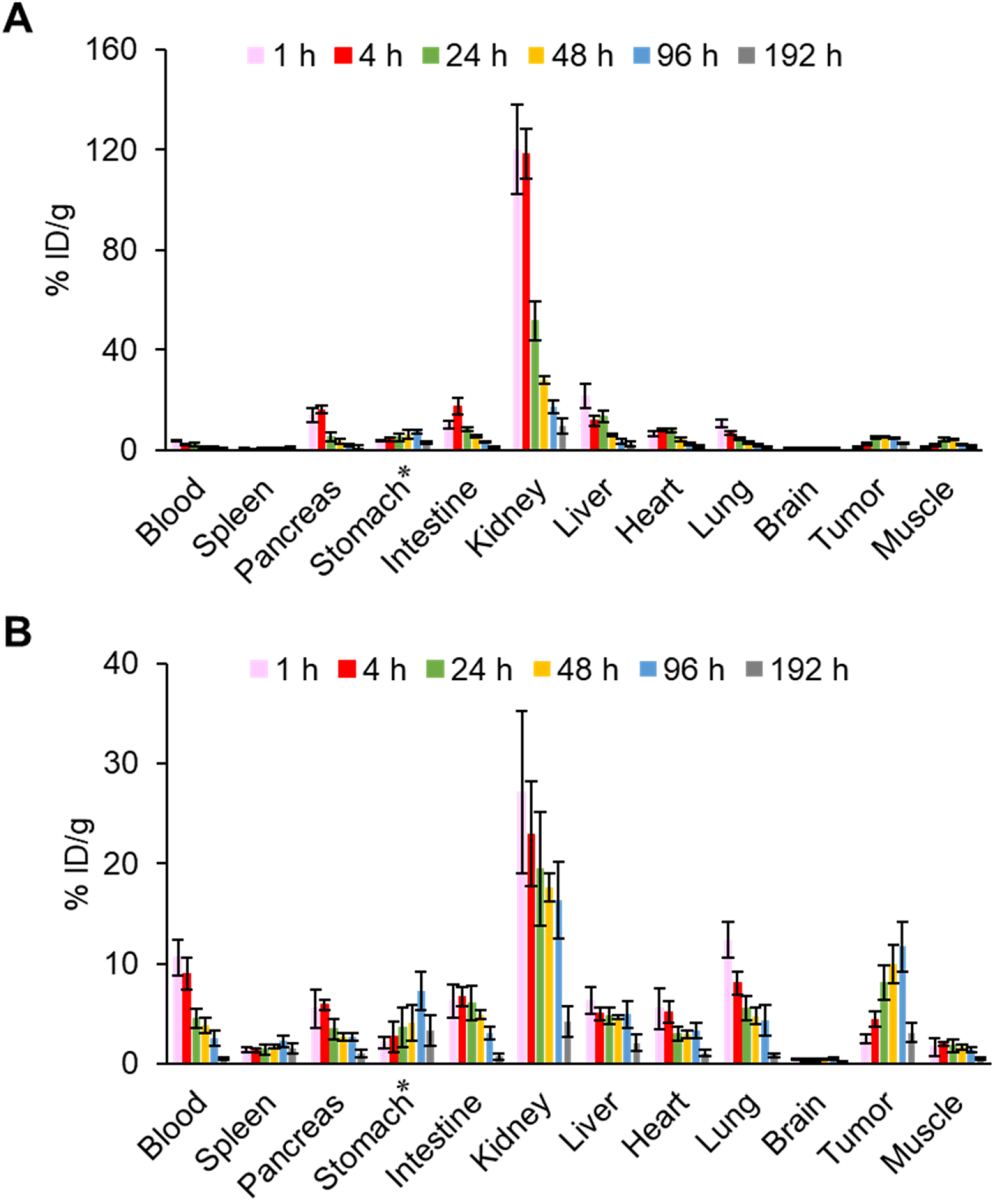
Biodistribution of radioactivity among organs and tissues after intravenous injection of IS-[^111^In]In-DOTADG-ALB **(A)** and [^111^In]In-DOTAGA-ALB-IS **(B)** into HT-29 tumor-bearing mice (*n* = 4). *Values are expressed as % ID.

**Figure 7:**
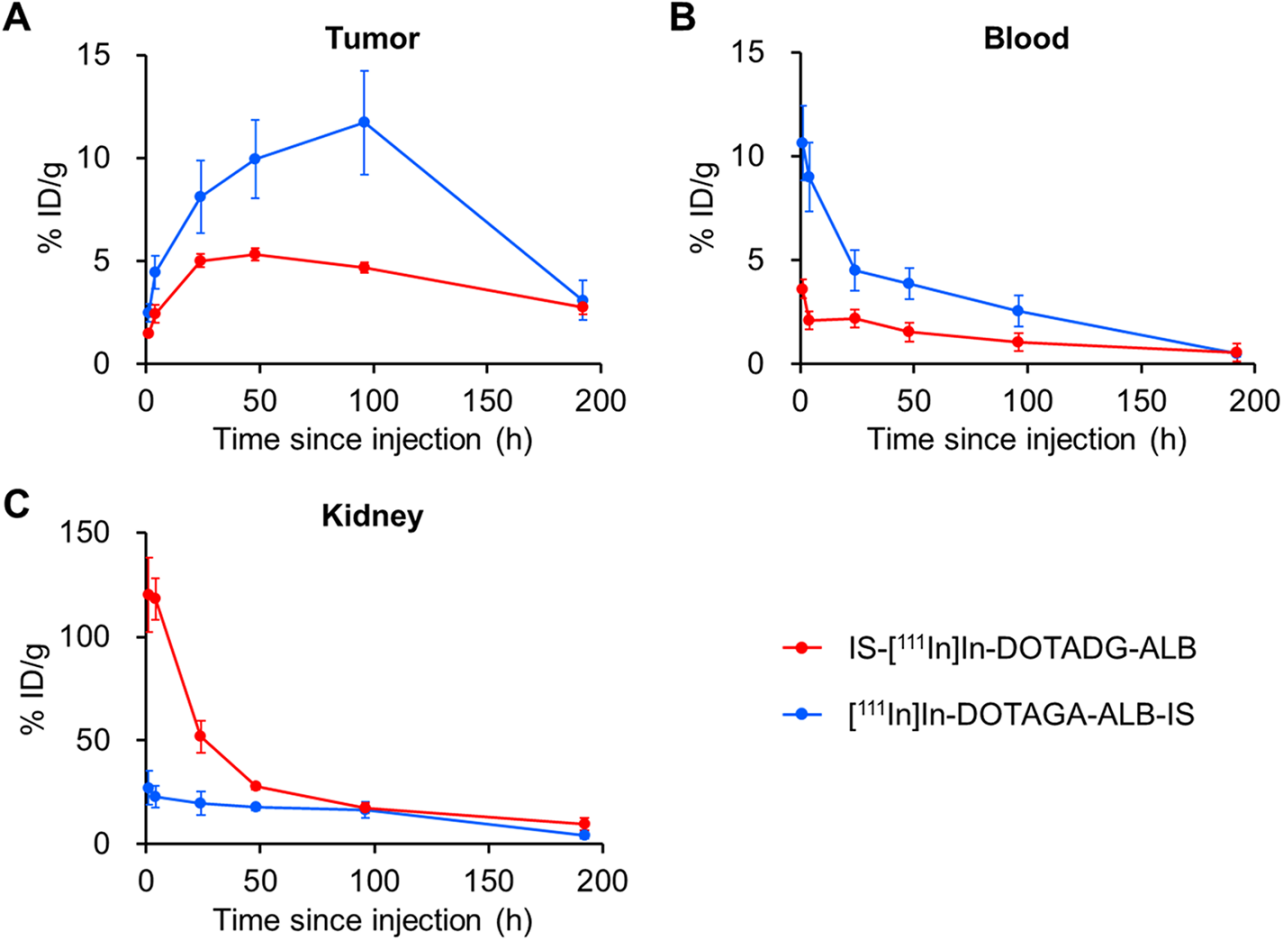
Comparison of radioactivity in the tumor **(A)**, blood **(B)**, and kidney **(C)** between IS-[^111^In]In-DOTADG-ALB and [^111^In]In-DOTAGA-ALB-IS.

The pharmacokinetics of IS-[^111^In]In-DOTADG-ALB and [^111^In]In-DOTAGA-ALB-IS were evaluated by a biodistribution assay using HT-29 tumor-bearing mice (Figure 6, Tables S1, S2). Both radioligands showed tumor accumulation after their intravenous injection, and the tumor accumulation of [^111^In]In-DOTAGA-ALB-IS was higher than that of IS-[^111^In]In-DOTADG-ALB at all time-points (Figure 7A). Regarding blood radioactivity, [^111^In]In-DOTAGA-ALB-IS showed marked retention, which was higher than that of IS-[^111^In]In-DOTADG-ALB (Figure 7B). Moreover, the renal uptake of IS-[^111^In]In-DOTADG-ALB reached up to 120.1 ± 17.7% ID/g at 1-h p.i.; however, the maximum renal uptake of [^111^In]In-DOTAGA-ALB-IS was only 27.11 ± 8.13% ID/g at 1-h p.i. (Figure 7C). These differences in pharmacokinetic profiles in the tumor, blood, and kidney suggest that Lys-IPBA of [^111^In]In-DOTAGA-ALB-IS may function more efficiently as ALB *in vivo* than that of IS-[^111^In]In-DOTADG-ALB, resulting in prolonged blood clearance, efficient tumor delivery, and avoidance of glomerular filtration in the kidney for [^111^In]In-DOTAGA-ALB-IS. This is supported by the results of the *in vitro* albumin-binding assay, which demonstrated the greater albumin-binding properties of [^111^In]In-DOTAGA-ALB-IS compared with IS-[^111^In]In-DOTADG-ALB.

In our previous study, IS-[^111^In]In-DO2A-ALB1 in a linear-chain arrangement exhibited marked albumin binding by the introduction of Lys-IPBA, resulting in enhanced tumor accumulation and reduced renal uptake (25), being similar to the pharmacokinetics of branched-chain [^111^In]In-DOTAGA-ALB-IS. However, this study indicated the impaired albumin-binding properties of Lys-IPBA in linear-chain IS-[^111^In]In-DOTADG-ALB. Considering that DOTADG-ALB-based radioligands targeting prostate-specific membrane antigen or glucagon-like peptide-1 receptor have shown favorable albumin-binding properties *in vitro* and *in vivo* (23, 37), the IS-DOTADG structure may interfere with the interaction of the ALB moiety with albumin for IS-[^111^In]In-DOTADG-ALB due to other than the blocking of the binding pocket of albumin, although the details remain unclear. These data suggest that a branched-chain arrangement is a favorable molecular design for CA-IX-targeted radioligands consisting of an IS ligand, DOTA, and Lys-IPBA.

## Conclusion

We developed CA-IX-targeted trifunctional radioligands, IS-[^111^In]In-DOTADG-ALB in a linear-chain arrangement and [^111^In]In-DOTAGA-ALB-IS in a branched-chain arrangement, consisting of an IS ligand to target CA-IX, DOTA to chelate radiometals, and Lys-IPBA as ALB to improve the pharmacokinetics. IS-[^111^In]In-DOTADG-ALB and [^111^In]In-DOTAGA-ALB-IS showed similar stabilities in murine plasma and affinities to CA-IX. Conversely, their affinities to albumin differed: [^111^In]In-DOTAGA-ALB-IS exhibited greater albumin-binding properties than IS-[^111^In]In-DOTADG-ALB. In the biodistribution study, [^111^In]In-DOTAGA-ALB-IS showed a higher tumor accumulation and lower renal uptake than IS-[^111^In]In-DOTADG-ALB, suggesting different pharmacokinetics of the radioligands based on their albumin-binding affinities modified by the arrangement of the IS ligand, DOTA, and Lys-IPBA. These data suggest that a branched-chain arrangement may be favorable for the design of CA-IX-targeted radioligands consisting of an IS ligand, DOTA, and Lys-IPBA.

## Data Availability

The raw data supporting the conclusions of this article will be made available by the authors, without undue reservation.
