# Supplementary material for: Comparison of carbonic anhydrase-IX-targeted trifunctional radioligands between linear- and branched-chain arrangements
Source: Front Nucl Med. 2025 Apr 16;5:1585027. doi: 10.3389/fnume.2025.1585027 (PMC12040898; doi:10.3389/fnume.2025.1585027)
Supplement: Supplementary file 1 [file Datasheet1.pdf]

Supplementary Material

Comparison of carbonic anhydrase-IX-targeted trifunctional radioligands between linear- and branched-chain arrangements

Kazuma Nakashima[1,†], Takayoshi Ichinose[1,†], Hiroyuki Watanabe[1], Masahiro Ono[1,*]

[1]*Department of Patho-Functional Bioanalysis, Graduate School of Pharmaceutical Sciences, Kyoto University, 46-29 Yoshida Shimoadachi-cho, Sakyo-ku, Kyoto 606-8501, Japan.*

[†]These authors have contributed equally to this work.

* Corresponding author

Masahiro Ono

Department of Patho-Functional Bioanalysis, Graduate School of Pharmaceutical Sciences, Kyoto University, 46-29 Yoshida Shimoadachi-cho, Sakyo-ku, Kyoto 606-8501, Japan; Tel.: +81-75-753-4556; Fax: +81-75-753-4568; E-mail: ono@pharm.kyoto-u.ac.jp

Table of Contents

Table S1. Biodistribution of radioactivity among organs and tissues after the intravenous injection of IS-[^{111}In]In-DOTADG-ALB into HT-29 tumor-bearing mice.......................S3

Table S2. Biodistribution of radioactivity among organs and tissues after the intravenous injection of [^{111}In]In-DOTAGA-ALB-IS into HT-29 tumor-bearing mice.......................S4

Figure S1. HPLC chromatograms of radioactivity and UV absorption in the purification of IS-[^{111}In]In-DOTADG-ALB..S5

Figure S2. HPLC chromatograms of radioactivity and UV absorption in the purification of [^{111}In]In-DOTAGA-ALB-IS..S5

Figure S3. HPLC chromatograms of radioactivity and UV absorption for a mixture of IS-[^{111}In]In-DOTADG-ALB and IS-[^{nat}In]In-DOTADG-ALB.......................................S6

Figure S4. HPLC chromatograms of radioactivity and UV absorption for a mixture of [^{111}In]In-DOTAGA-ALB-IS and [^{nat}In]In-DOTAGA-ALB-IS.......................................S6

Table S1. Biodistribution of radioactivity among organs and tissues after the intravenous injection of IS-[^{111}In]In-DOTADG-ALB into HT-29 tumor-bearing mice[a]

		Time since injection (h)					
		**4**	**24**	**48**	**72**	**96**	**192**
% ID/g	Blood	3.61 ± 0.30	2.09 ± 0.23	2.18 ± 0.88	1.53 ± 0.19	1.04 ± 0.24	0.54 ± 0.23
	Spleen	0.66 ± 0.11	0.53 ± 0.07	0.81 ± 0.16	0.91 ± 0.11	0.88 ± 0.23	1.22 ± 0.49
	Pancreas	13.95 ± 2.57	16.15 ± 1.51	5.32 ± 1.63	3.48 ± 0.85	1.87 ± 0.40	1.20 ± 0.63
	Stomach[b]	3.77 ± 0.34	4.31 ± 0.91	5.20 ± 1.60	6.16 ± 1.85	7.36 ± 0.70	2.97 ± 0.70
	Intestine	10.18 ± 1.48	17.46 ± 3.35	8.35 ± 0.56	5.71 ± 0.50	3.29 ± 0.18	1.31 ± 0.42
	Kidney	120.1 ± 17.7	118.3 ± 10.0	51.80 ± 7.86	27.91 ± 1.45	17.42 ± 2.64	9.71 ± 3.08
	Liver	21.58 ± 4.95	11.69 ± 2.09	13.25 ± 2.30	6.26 ± 0.45	3.48 ± 0.94	2.43 ± 1.01
	Heart	6.53 ± 1.16	8.17 ± 0.41	7.71 ± 0.83	4.41 ± 0.68	2.57 ± 0.44	1.64 ± 0.56
	Lung	10.71 ± 1.39	6.61 ± 0.75	4.51 ± 0.51	2.85 ± 0.58	2.01 ± 0.57	1.15 ± 0.47
	Brain	0.67 ± 0.05	0.78 ± 0.06	0.77 ± 0.09	0.98 ± 0.06	1.00 ± 0.08	0.81 ± 0.06
	Tumor	1.48 ± 0.14	2.41 ± 0.43	5.01 ± 0.33	5.31 ± 0.28	4.69 ± 0.25	2.76 ± 0.36
	Muscle	1.34 ± 0.22	2.07 ± 0.36	4.09 ± 0.79	4.15 ± 0.21	2.44 ± 0.20	1.45 ± 0.59
Ratio	Tumor/ Blood	0.41 ± 0.02	1.18 ± 0.29	2.70 ± 1.09	3.52 ± 0.35	4.68 ± 0.77	5.75 ± 0.65
	Tumor/ Muscle	1.12 ± 0.13	1.17 ± 0.08	1.25 ± 0.16	1.28 ± 0.04	1.93 ± 0.06	2.17 ± 0.65

[a]Each value represents the mean ± standard deviation of four mice.

[b]Data are expressed as % injected dose (ID) values.

Table S2. Biodistribution of radioactivity among organs and tissues after the intravenous injection of [^{111}In]In-DOTAGA-ALB-IS into HT-29 tumor-bearing mice[a]

		Time since injection (h)					
		**4**	**24**	**48**	**72**	**96**	**192**
% ID/g	Blood	10.62 ± 1.80	8.99 ± 1.65	4.52 ± 0.98	3.86 ± 0.76	2.53 ± 0.74	0.49 ± 0.16
	Spleen	1.43 ± 0.27	1.34 ± 0.16	1.40 ± 0.48	1.67 ± 0.18	2.27 ± 0.57	1.51 ± 0.56
	Pancreas	5.52 ± 1.90	5.88 ± 0.51	3.45 ± 1.05	2.63 ± 0.38	2.65 ± 0.42	1.00 ± 0.34
	Stomach[b]	2.10 ± 0.53	2.72 ± 1.53	3.62 ± 2.03	4.08 ± 1.76	7.28 ± 1.95	3.35 ± 1.54
	Intestine	6.25 ± 1.72	6.70 ± 0.98	6.08 ± 1.70	4.90 ± 0.41	3.07 ± 0.62	0.71 ± 0.33
	Kidney	27.11 ± 8.13	22.98 ± 5.18	19.47 ± 5.68	17.64 ± 1.39	16.38 ± 3.85	4.20 ± 1.54
	Liver	6.29 ± 1.35	4.98 ± 0.60	4.83 ± 0.82	4.62 ± 0.19	4.92 ± 1.35	2.05 ± 0.82
	Heart	5.49 ± 2.01	5.16 ± 1.03	2.99 ± 0.69	2.92 ± 0.40	3.31 ± 0.80	1.06 ± 0.35
	Lung	12.36 ± 1.75	8.05 ± 1.18	5.49 ± 1.21	4.75 ± 0.81	4.34 ± 1.54	0.83 ± 0.21
	Brain	0.42 ± 0.07	0.38 ± 0.07	0.39 ± 0.08	0.40 ± 0.05	0.51 ± 0.12	0.22 ± 0.05
	Tumor	2.46 ± 0.44	4.43 ± 0.80	8.10 ± 1.75	9.94 ± 1.89	11.71 ± 2.52	3.09 ± 0.96
	Muscle	1.70 ± 0.90	1.98 ± 0.19	1.79 ± 0.62	1.64 ± 0.20	1.36 ± 0.28	0.47 ± 0.12
Ratio	Tumor/Blood	0.23 ± 0.02	0.50 ± 0.05	1.81 ± 0.28	2.58 ± 0.20	4.71 ± 0.36	6.36 ± 0.84
	Tumor/Muscle	1.70 ± 0.48	2.23 ± 0.33	4.85 ± 1.01	6.15 ± 1.26	8.69 ± 0.91	6.51 ± 0.84

[a]Each value represents the mean ± standard deviation of four mice.

[b]Data are expressed as % ID values.

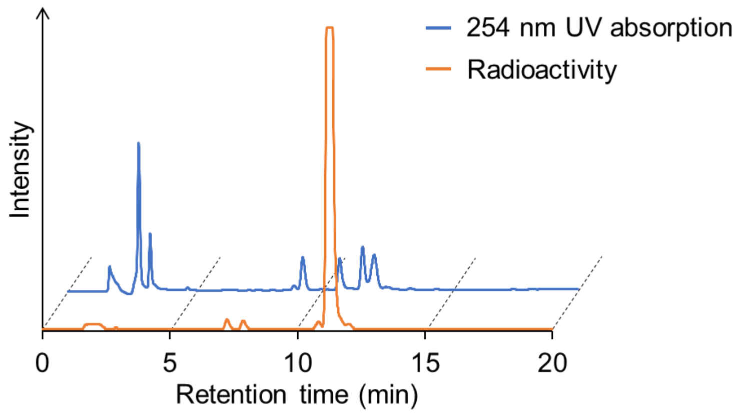

Figure S1. HPLC chromatograms of radioactivity and UV absorption at 254 nm in the purification of IS-[^{111}In]In-DOTADG-ALB.

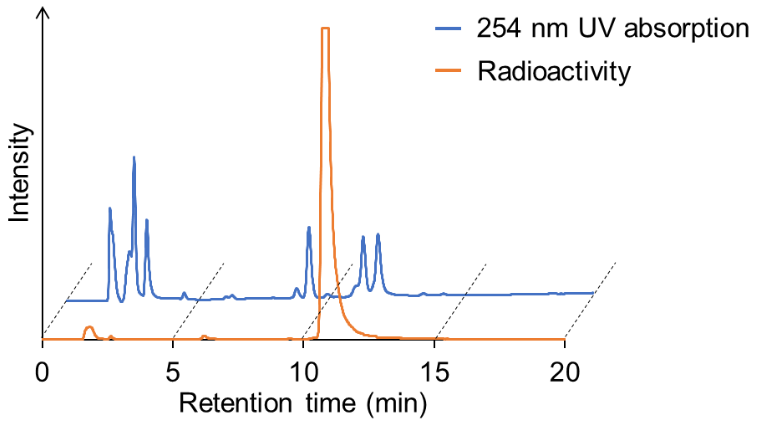

Figure S2. HPLC chromatograms of radioactivity and UV absorption at 254 nm in the purification of [^{111}In]In-DOTAGA-ALB-IS.

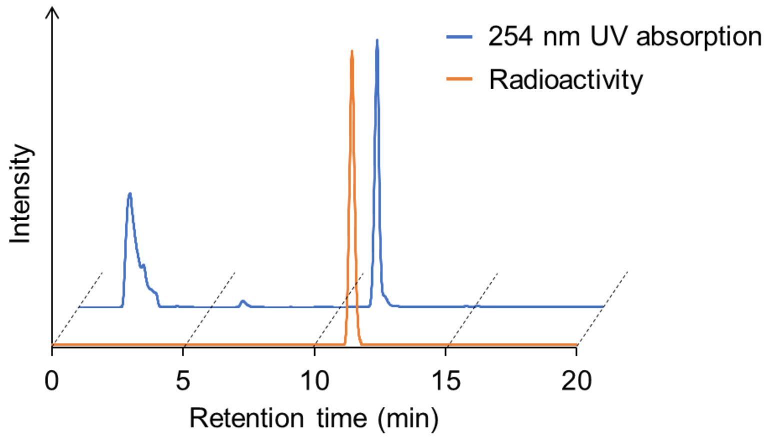

Figure S3. HPLC chromatograms of radioactivity and UV absorption at 254 nm for a mixture of IS-[^{111}In]In-DOTADG-ALB and IS-[^{nat}In]In-DOTADG-ALB. The detected UV absorption around 2-min postinjection is considered to be derived from DMSO.

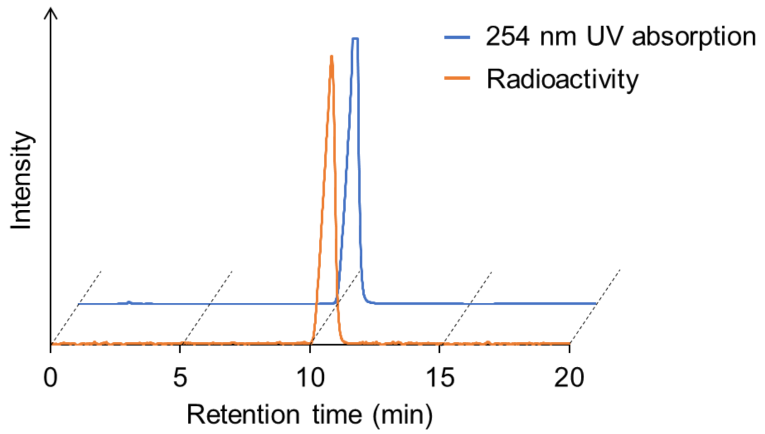

Figure S4. HPLC chromatograms of radioactivity and UV absorption at 254 nm for a mixture of [^{111}In]In-DOTAGA-ALB-IS and [^{nat}In]In-DOTAGA-ALB-IS.
